# Rural Morality

**Published:** 1915-08

**Authors:** 

**Affiliations:** San Antonio


### Rural Morality.

There is, perhaps, some real interest in knowing whether the city or the country is the most moral. At any rate, the opinion prevails that immorality is the most prevalent in the rural districts. Dr. Paul L. Vogt, professor of sociology, Miami University, has recently made a survey of this subject for *Social Hygiene,* and the conclusions arrived at are of use to all social workers. However, it must be noted that the idea "moral," as used in this report, is the same as our traditional conception of this term and the report is of value only as it relates to this standard.

Morality of any community is the result of social heredity rather than organic heredity and therefor more plastic and subject to greater changes. So long as society is an active agency there must be changes, and, if it is to endure, this change must be in the direction of race betterment. The standard, therefore, for morality is whether customs and habits of the people afford for them the greatest happiness and benefit. In our study of eugenics it is becoming evident that some of our ideas of morality will be changed, and a new standard of conduct established for our guidance. For instance, we have in the past outlived equally imperative belief in witchcraft, the inquisition, religious persecution, subjugation of woman, slavery, and we are a good way along towards the abolishment of war.

The doctor chose for his study two representative districts and his inquiry embraced: "(1) The number of cases of venereal diseases treated during the past year by the local physicians in the two counties studied. These data were obtained according to the different important types, according to the sex, conjugal condition, whether the case was local (i. e., within the county), or outside the community, and as far as possible according to the source of infection; (2) evidence of forced marriages as shown by comparisons of marriage date with records of birth of first child; (3) extent of illegitimacy; (4) records of juvenile courts; (5) records of criminal cases in the county and before mayors and justices of the peace; (6) divorce records, classified according to residence in village or open country."

And he concludes: "The data shows that the village is in many ways the central point in social life outside the cities. It appears that the larger the aggregation of people the more serious the problems of social pathology become. Disease, divorce, and delinquency thrive in the village. Yet this is but in part true. The type of vice changes. The vices of the open country are those of isolation. There are to be found sordid, illicit, incestuous relations free from disease only because contact with sources of disease has not been possible. There thrive the crude, rowdy, fighting elements. Bringing people together and increasing facilities of transportation brings culture and lessens the vices of isolation, but substitutes those of congregation. The great problem for the church and the school is to take control of the recreational life of the village so that there will be an abundance of wholesome social and recreational contact under proper auspices and under favorable conditions. There is need that both girls and boys, men and women know more of the significance of vice and its effect upon themselves and the community; know more about what real uplifting enjoyment means; learn to hate the evil and love the good; learn more of the meaning of the family and more of what is necessary to make family life helpful and happy. Both the preacher and the teacher need to emphasize more and more the principles of living in modern society and to take the lead in eliminating evil by substituting an abundance of good."

It is obvious that in gathering statistics, bearing on moral subjects, there is much of the essential lacking. The individual will not confide his secret life and all the information that can be secured is in cases of flagrant violation of the accepted standard. "By their fruits ye shall know them," and so it is by the manifestations observable that any community can be rightly judged.

---

Seldom is a slovenly doctor successful. Personal neatness and good address count more with the feminine clientele than some matters of really greater importance.—*Medical Summary.*

---

### The Doctor Himself.

Like his rooms and attendants, the doctor himself should be absolutely clean, both as to his person and clothing. During the hours of consultation he should be clothed in white, preferably. While there is some expense attached to the wearing of white clothing, the appearance of the doctor more than balances this. He who comes to be known as "the doctor in white" not infrequently adds to his reputation. The secret is that dirt shows on clothing of this sort, and he with a dirty white suit never looks neat or inviting to the patient.

It is not necessary that the doctor wear a gown in his consultation room. A pair of white duck trousers and an army style white blouse are the essentials. As the blouse is worn buttoned to the chin, the doctor may wear his customary waistcoat underneath. if he so desires. His linen should be scrupulously clean, and his shirts should be made with the soft French cuffs, so that he may be able to roll his sleeves up easily. To carry out the white scheme in its entirety, white shoes should be added, those with rubber soles preferably.

The doctor's hands should be well manicured. We do not mean by this that his finger nails should be tinted and polished, but they should be well trimmed and clean. Nothing offends the patient as does an accumulation of dirt underneath the nails of the doctor. Such a condition arouses the curiosity of the patient and makes him wonder if the doctor is as careless in other particulars. As the doctor depends largely upon the sense of touch in much of his work, his hands should be kept soft and free from chap, scratches or injury. As he has his hands much in water, he should dry them thoroughly and apply some emolient after getting them wet.—*Physician Drug News.*

